# Magnetic resonance imaging predictors (cartilage, osteophytes and meniscus) of prevalent and 3-year incident medial and lateral tibiofemoral knee joint tenderness and patellofemoral grind

**DOI:** 10.1186/s12891-022-06033-x

**Published:** 2022-12-02

**Authors:** Eric C. Sayre, Ali Guermazi, Savvas Nicolaou, John M. Esdaile, Jacek A. Kopec, Joel Singer, Hubert Wong, Anona Thorne, Jolanda Cibere

**Affiliations:** 1 Arthritis Research Canada, 230-2238 Yukon Street, Vancouver, BC V5Y 3P2 Canada; 2 Radiology, Boston University School of Medicine, Boston, MA USA; 3 Radiology, University of British Columbia, Vancouver, BC Canada; 4 Medicine, University of British Columbia, Vancouver, BC Canada; 5 Medicine, University of Calgary, Calgary, AB Canada; 6 School of Population and Public Health, University of British Columbia, Vancouver, BC Canada

**Keywords:** Osteoarthritis (OA), Magnetic resonance imaging (MRI), Medial tibiofemoral tenderness, Lateral tibiofemoral tenderness, Patellofemoral grind

## Abstract

**Objective:**

To identify magnetic resonance imaging (MRI) predictors (cartilage [C], osteophytes [O] and meniscus [M] scores) of prevalent and 3-year incident medial tibiofemoral (MTF) and lateral tibiofemoral (LTF) knee joint tenderness and patellofemoral (PF) grind.

**Methods:**

Population-based knee pain cohort aged 40–79 was assessed at baseline (*N* = 255), 3- and 7-year follow-up (*N* = 108 × 2 = 216). COM scores were measured at 6/8/6 subregions respectively. Age-sex-BMI adjusted logistic models predicted prevalence versus relevant COM predictors (medial, lateral or patellar / trochlear groove scores). Fully adjusted models also included all relevant COM predictors. Binary generalized estimating equations models predicting 3-year incidence were also adjusted for individual follow-up time between cycles.

**Results:**

Significant predictors of prevalent MTF tenderness: medial femoral cartilage (fully adjusted odds ratio [aOR] 1.84; 95% confidence interval [CI] 1.11, 3.05), female (aOR = 3.05; 1.67, 5.58), BMI (aOR = 1.53 per 5 units BMI; 1.10, 2.11). Predictors of prevalent LTF tenderness: female (aOR = 2.18; 1.22, 3.90). There were no predictors of prevalent PF grind in the fully adjusted model. However, medial patellar osteophytes was predictive in the age-sex-BMI adjusted model. There were no predictors of 3-year incident MTF tenderness. Predictors of 3-year incident LTF tenderness: female (aOR = 3.83; 1.25, 11.77). Predictors of 3-year incident PF grind: lateral patellar osteophytes (aOR = 4.82; 1.69, 13.77). In the age-sex-BMI adjusted model, patellar cartilage was also a predictor.

**Conclusion:**

We explored potential MRI predictors of prevalent and 3-year incident MTF/LTF knee joint tenderness and PF grind. These findings could guide preemptive strategies aimed at reducing these symptoms in the present and future (3-year incidence).

## Introduction

Osteoarthritis (OA) is a highly prevalent, disabling and costly disease which may occur in smaller joints such as hands, but most often manifests in the larger weight-bearing joints including knees and hips [1, 2]. Knee OA is characterized by structural changes in cartilage (either detectable on X-ray as joint space narrowing or on magnetic resonance imaging [MRI] with greater specificity), bone (e.g., osteophytes) and/or meniscal damage, and symptoms and signs may include joint pain, stiffness, tenderness, crepitus, limitation of movement and effusion [3]. Diagnosis can be made on the basis of radiographic evidence of structural defects without symptoms (radiographic OA), radiographic evidence with pain or other symptoms (symptomatic radiographic OA), or oftentimes can be made on the basis of clinical examination [4]. Using nationally representative data from the 2015 Medical Expenditure Panel Survey, the self-reported prevalence of OA among noninstitutionalized U.S. adults was estimated at 10.5% (25.6 million) [1]. OA is more prevalent with older age and in obese people and thus, OA constitutes an increasing public health burden. In the U.S., the adjusted incremental annual healthcare cost and wage loss among adults with self-reported OA vs. those without OA resulted in an estimated national excess cost totaling $46.7 billion in 2015 [1].

In addition to radiographic assessments of structural knee OA such as the previously de facto coarse-grained Kellgren-Lawrence (KL) grade, [5] or modern MRI measures of structural OA such as the fine-grained OA-COM scoring system, [6] various clinical assessments/signs of OA are investigated when determining OA. Clinical signs of OA include (among others) medial and lateral tibiofemoral (MTF/LTF) tenderness and patellofemoral (PF) grind [7–9]. Tenderness (pain on palpation of a specific part of the joint) and PF grind are not well understood with respect to structural defects [10]. Structural defects can coincide with, or in some cases precede by years the onset of physical symptoms such as tenderness or grind. Developing a better understanding of how structural changes due to OA are associated with present or future symptoms could help guide preemptive strategies aimed at reducing the prevalence or incidence of those symptoms, and therefore presents a valuable research question. While the literature is somewhat deeper on the question of structural correlates of pain on movement in knee OA, there have been relatively few studies investigating structural correlates of knee joint tenderness (on palpation) or PF grind, and the structural measures in these studies were generally aggregated, using for example such aggregated measures as “tibiofemoral OA” or “patellofemoral OA” [7]. However, pertinent structural defects related to OA may occur in various specific structures (e.g., cartilage/osteophytes/meniscus) and within various specific compartments (medial/lateral, anterior/posterior, tibiofemoral/patellofemoral/trochlear groove). The data utilized in our study include such specificity. Improving our understanding of the structure-symptom relationship by analyzing these lesser studied symptoms versus highly specific structural variables would be immediately beneficial to OA researchers, and in future, could also help guide clinical interventions aimed at reducing symptomatic disease [10].

The purpose of this study is two-fold. First, we seek to elucidate which (if any) MRI knee joint scores on cartilage, osteophytes and/or meniscal damage within a multitude of subregions are associated cross-sectionally with prevalent MTF/LTF knee joint tenderness and/or PF grind. Secondly, we explore structural predictors of 3-year incident (new onset) tenderness and grind.

## Materials and methods

### Ethics approval

This study was conducted in accordance with the declaration of Helsinki and was approved by the Clinical Research Ethics Board of the University of British Columbia. All participants gave written informed consent at all three time points.

### Data collection

Source data came from a longitudinal study conducted in Vancouver, Canada, [11] a population-based cohort of individuals aged 40 to 79 with knee pain “on most days of the month at any time in the past and any pain in the past 12 months.” Data collection has been previously described [12, 13]. The clinical examination was performed by an experienced rheumatologist (JC). We have previously reported in this cohort that, based on MRI cartilage damage and X-ray findings, 13% had no OA (KL < 2 and no cartilage damage), 49% had pre-radiographic OA (cartilage damage but KL < 2), and 38% had radiographic OA (KL ≥ 2) [13]. This cohort enrolled 255 individuals, stratified by age decade and sex in roughly equal group sizes to ensure adequate sample size across the age-sex spectrum [14]. Baseline visits occurred between 2002 and 2005. In addition to the baseline cycle, two follow-up cycles were undertaken, at stratum-sampling-weighted mean 3.3 (SD 0.6) and 7.5 (SD 0.6) years. The present study uses the baseline sample (*N* = 255) for the cross-sectional modelling, as well as the intersection of the first and second follow-up cycles (*N* = 108 × 2 = 216) for longitudinal modelling.

The study knee was the more painful knee at baseline. X-rays were obtained using a weight-bearing fixed-flexion posteroanterior view with the SynaFlexer (BioClinica Inc., Newark, CA, USA) positioning frame, and a skyline view in the supine position [15]. Radiographs were read blinded to clinical information by two independent readers for KL 0–4 grading [5]. Previous studies using these data have demonstrated good interrater reliability (ICC = 0.79) [12]. Differences in readings were adjudicated by consensus readings with both readers. MRIs were acquired on a GE 1.5 T magnet at a single centre using a transmitter–receiver extremity knee coil. The imaging protocol included four MRI sequences, as previously described [13, 14]. MRIs were scored by a board-certified musculoskeletal radiologist (AG) who was blinded to clinical, radiographic, and time sequence information. Cartilage was scored in 6 subregions: lateral and medial femur, lateral and medial tibia, patella and trochlear groove. The trochlear groove was delineated from the weight bearing surfaces of the femur by oblique lines, tangent to the anterior tips of the anterior horns of the medial and lateral menisci [14]. Cartilage was graded on a 0–4 semi-quantitative scale based on the following definitions, previously described by Disler et al.: [16] 0: normal, 1: abnormal signal without cartilage contour defect, 2: contour defect of < 50% cartilage thickness, 3: contour defect of 50–99% cartilage thickness, 4: 100% cartilage contour defect with subjacent bone signal abnormality. 0 and 1 were collapsed since 1 represents signal hyperintensity on T2-weighted images of indeterminate significance, hence the analysis variables ranged from 0–3. Osteophytes (defined as osteo-cartilaginous protrusions growing at the margins of osteoarthritic joints from a process that involves endochondral ossification) were scored using criteria described in Hunter et al [17]. Osteophytes (0: absent, 1: small, 2: moderate, 3: large) were scored in 8 regions: lateral and medial femur, lateral and medial tibia, and lateral, medial, superior and inferior patella. Meniscal damage was scored as: 0: normal, 1: intra-substance signal, 2: tear. 0 and 1 were collapsed, hence the analysis variables ranged from 0–1. Meniscal damage was scored in the following 6 regions: lateral anterior, lateral body, lateral posterior, medial anterior, medial body and medial posterior. Intra-rater reliability analyses were previously performed on the scoring of each surface within each feature. The ranges of intraclass correlation coefficients (ICCs) across regions were: cartilage 0.84–1.00, osteophytes 0.77–0.89, meniscus 0.60–0.83 [8].

MTF tenderness (ICC = 0.94) and LTF tenderness (ICC = 0.85) were assessed in examination by palpating the medial or lateral tibiofemoral joint line while the patient sits with legs hanging over the edge of the examination bed. PF grind (ICC = 0.94) was assessed with the subject lying on the examination bed with their legs extended, then asked to contract their quadriceps muscle while the examiner applies downward and inferior pressure on the patella. Pain was the positive signal in both tests (not grinding).

### Statistical methods

To obtain population-representative results, a baseline sample weight was developed as the ratio of knee-pain population age-sex distribution over the baseline knee-pain sample distribution, and was used in the cross-sectional models. Cross-sectional (prevalence) models were weighted with the baseline sample weight. A sample weight was developed for the longitudinal sample as the baseline sample weight multiplied by the ratio of baseline sample proportion in a given age-sex cell over the longitudinal sample proportion in that cell. Longitudinal (incidence) models were weighted with the longitudinal sample weight.

For our first objective (modelling cross-sectional association), we used baseline data to fit age-sex-BMI adjusted logistic models predicting prevalent MTF and LTF knee joint tenderness as well as PF grind versus each relevant cartilage/osteophyte/meniscus (COM) predictor in separate models. Relevant COM predictors for MTF tenderness included medial femoral cartilage (MFC), medial tibial cartilage (MTC), medial femoral osteophytes (MFO), medial tibial osteophytes (MTO), medial anterior meniscus (MAM), medial meniscal body (MBM), and medial posterior meniscus (MPM). The relevant COM predictors for LTF tenderness included the lateral equivalents of those medial predictors. The relevant COM predictors for PF grind included patellar cartilage (PC), trochlear groove cartilage (TC), medial patellar osteophytes (MPO), lateral patellar osteophytes (LPO), superior patellar osteophytes (SPO) and inferior patellar osteophytes (IPO). In addition to the age-sex-BMI adjusted single COM predictor models, we also fit fully adjusted multivariable models including as predictors all relevant COM predictors together, plus age, sex and BMI. For our second objective (modelling 3-year incidence), we combined baseline to 3-year follow-up with 3-year to 7-year follow-up (two records per subject), and fit binary generalized estimating equations (GEE) models predicting 3-year incident MTF and LTF knee joint tenderness as well as 3-year incident PF grind versus the relevant COM predictors at “baseline” for each cycle (i.e., either actual baseline or 3 years depending on the cycle represented on a given record). Incidence models were also fit as age-sex-BMI adjusted single COM predictor models, as well as fully adjusted models including as predictors all relevant COM predictors together, plus age, sex and BMI. All longitudinal models were also adjusted for individual follow-up time between cycles. Model fit was assessed via the Hosmer and Lemeshow goodness of fit test [18]. The predictive utility of each model was assessed via the area under the receiver operating characteristic (ROC) curve (AUC).

Due to theoretical considerations (lack of a plausible biological pathway), in the primary models we did not regress PF grind versus medial or lateral tibiofemoral MRI scores. However, in a sensitivity analysis, we fit models predicting PF grind versus each of the medial and lateral predictor sets listed above.

Analyses were performed using SAS version 9.4 (SAS Institute Inc., Cary, NC, USA).

## Results

Table 1 describes the baseline predictors and covariates for cross-sectional and longitudinal analyses, respectively weighted *N* = 255.0 and 217.3 (2 times the weighted intersection between cycles 2 and 3). In the cross-sectional sample, weighted *n* = 143.6 (56.3%) were female, weighted mean age was 56.7 (standard deviation [SD] = 10.4) and mean BMI was 26.5 (SD = 4.9). Radiographic OA (defined as KL grade ≥ 2) was observed in 98.0 (38.4%) subjects. Abnormal grades (> 0) were observed for MFC at 66.5%, MTC at 50.5%, MFO at 61.7%, MTO at 79.0%, MAM at 4.3%, MBM at 25.4%, MPM at 33.5%, LFC at 57.8%, LTC at 25.6%, LFO at 51.0%, LTO at 69.2%, LAM at 8.5%, LBM at 8.3%, LPM at 5.9%, PC at 61.5%, TC at 52.4%, MPO at 47.6%, LPO at 73.1%, SPO at 86.2% and IPO at 38.6%. In the longitudinal sample, 119.8 (55.1%) were female, mean age was 57.2 (SD = 9.0) and mean BMI was 26.1 (SD = 4.2). Radiographic OA was observed in 99.2 (45.7%) subjects. Abnormal grades (> 0) were observed for MFC at 62.7%, MTC at 44.8%, MFO at 64.9%, MTO at 65.6%, MAM at 9.5%, MBM at 28.7%, MPM at 38.3%, LFC at 33.8%, LTC at 37.5%, LFO at 91.8%, LTO at 75.4%, LAM at 8.7%, LBM at 14.4%, LPM at 11.4%, PC at 60.8%, TC at 51.4%, MPO at 73.7%, LPO at 86.4%, SPO at 88.6% and IPO at 56.1%.

**Table 1:** Baseline predictors and covariates for cross-sectional and longitudinal analyses, n (%) or mean (SD)

Variable	Cross-sectional (wgt. *N* = 255.0)	Longitudinal (wgt. *N* = 217.3)
Female	143.6 (56.3)	119.8 (55.1)
Age, mean (SD)	56.7 (10.4)	57.2 (9.0)
BMI, mean (SD)	26.5 (4.9)	26.1 (4.2)
KL grade		
0	104.9 (41.1)	70.4 (32.4)
1	52.1 (20.4)	47.7 (22.0)
2	48.7 (19.1)	52.0 (23.9)
3	26.9 (10.6)	24.3 (11.2)
4	22.3 (8.8)	22.9 (10.5)
Medial femoral cartilage		
0	85.3 (33.5)	81.0 (37.3)
1	91.4 (35.9)	72.5 (33.4)
2	49.1 (19.3)	36.8 (16.9)
3	29.2 (11.4)	26.9 (12.4)
Medial tibial cartilage		
0	126.1 (49.5)	119.9 (55.2)
1	75.3 (29.5)	52.2 (24.0)
2	27.3 (10.7)	20.9 (9.6)
3	26.3 (10.3)	24.3 (11.1)
Medial femoral osteophytes		
0	97.7 (38.3)	76.3 (35.1)
1	80.5 (31.6)	103.6 (47.7)
2	42.1 (16.5)	23.3 (10.7)
3	34.7 (13.6)	14.1 (6.5)
Medial tibial osteophytes		
0	53.5 (21.0)	74.7 (34.4)
1	125.9 (49.4)	128.5 (59.1)
2	36.1 (14.1)	11.1 (5.1)
3	39.5 (15.5)	3.0 (1.4)
Medial anterior meniscus		
0	244.2 (95.8)	196.6 (90.5)
1	10.8 (4.3)	20.7 (9.5)
Medial meniscal body		
0	190.3 (74.6)	154.8 (71.3)
1	64.7 (25.4)	62.5 (28.7)
Medial posterior meniscus		
0	169.7 (66.5)	134.1 (61.7)
1	85.3 (33.5)	83.2 (38.3)
Lateral femoral cartilage		
0	107.7 (42.2)	143.8 (66.2)
1	79.6 (31.2)	36.2 (16.7)
2	40.1 (15.7)	22.8 (10.5)
3	27.6 (10.8)	14.4 (6.7)
Lateral tibial cartilage		
0	189.8 (74.4)	135.8 (62.5)
1	30.7 (12.1)	46.9 (21.6)
2	17.7 (7.0)	15.1 (6.9)
3	16.8 (6.6)	19.5 (9.0)
Lateral femoral osteophytes		
0	124.9 (49.0)	17.7 (8.2)
1	66.9 (26.3)	122.1 (56.2)
2	31.3 (12.3)	69.5 (32.0)
3	31.9 (12.5)	7.9 (3.6)
Lateral tibial osteophytes		
0	78.4 (30.8)	53.5 (24.6)
1	95.0 (37.3)	124.5 (57.3)
2	45.2 (17.7)	38.2 (17.6)
3	36.3 (14.3)	1.1 (0.5)
Lateral anterior meniscus		
0	233.5 (91.6)	198.4 (91.3)
1	21.5 (8.5)	18.8 (8.7)
Lateral meniscal body		
0	233.8 (91.7)	186.1 (85.6)
1	21.2 (8.3)	31.2 (14.4)
Lateral posterior meniscus		
0	240.1 (94.2)	192.5 (88.6)
1	14.9 (5.9)	24.8 (11.4)
Patellar cartilage		
0	98.2 (38.5)	85.1 (39.2)
1	73.1 (28.7)	71.9 (33.1)
2	59.0 (23.2)	43.2 (19.9)
3	24.7 (9.7)	17.2 (7.9)
Trochlear groove cartilage		
0	121.4 (47.6)	105.6 (48.6)
1	62.6 (24.5)	67.5 (31.1)
2	57.1 (22.4)	34.6 (15.9)
3	13.9 (5.5)	9.6 (4.4)
Medial patellar osteophytes		
0	133.5 (52.4)	57.2 (26.3)
1	88.2 (34.6)	115.4 (53.1)
2	26.3 (10.3)	41.1 (18.9)
3	7.0 (2.8)	3.6 (1.7)
Lateral patellar osteophytes		
0	68.6 (26.9)	29.6 (13.6)
1	117.0 (45.9)	151.4 (69.7)
2	47.2 (18.5)	33.3 (15.3)
3	22.1 (8.7)	3.0 (1.4)
Superior patellar osteophytes		
0	35.1 (13.8)	24.8 (11.4)
1	161.2 (63.2)	144.3 (66.4)
2	39.6 (15.5)	42.8 (19.7)
3	19.1 (7.5)	5.4 (2.5)
Inferior patellar osteophytes		
0	156.6 (61.4)	95.3 (43.9)
1	63.6 (24.9)	102.8 (47.3)
2	22.1 (8.7)	19.2 (8.9)
3	12.7 (5.0)	0.0 (0.0)

Table 2 lists the prevalence of medial/lateral tibiofemoral tenderness and patellofemoral grind at baseline, 3 and 7 years, respectively weighted N = 255.0, 108.6 and 108.6. At baseline, medial tibiofemoral tenderness was observed in 143.7 (56.4%) subjects, lateral tibiofemoral tenderness in 77.8 (30.5%) subjects, and patellofemoral grind in 54.5 (21.4%) subjects. At 3 years, medial tibiofemoral tenderness was observed in 47.2 (43.5%) subjects, lateral tibiofemoral tenderness in 27.2 (25.1%) subjects, and patellofemoral grind in 15.2 (14.0%) subjects. At 7 years, medial tibiofemoral tenderness was observed in 44.7 (41.1%) subjects, lateral tibiofemoral tenderness in 17.2 (15.8%) subjects, and patellofemoral grind in 14.8 (13.6%) subjects.

**Table 2:** Medial/lateral tibiofemoral tenderness and patellofemoral grind at baseline, 3 and 7 years, n (%)

Variable	Baseline (wgt. *N* = 255.0)	3 years (wgt. *N* = 108.6)	7 years (wgt. *N* = 108.6)
Medial tibiofemoral tenderness	143.7 (56.4)	47.2 (43.5)	44.7 (41.1)
Lateral tibiofemoral tenderness	77.8 (30.5)	27.2 (25.1)	17.2 (15.8)
Patellofemoral grind	54.5 (21.4)	15.2 (14.0)	14.8 (13.6)

Table 3 lists the cross-sectional (prevalence) model odds ratios with 95% confidence intervals (CIs), both age-sex-BMI adjusted as well as fully adjusted. In the fully adjusted model, significant predictors of MTF tenderness included MFC (fully adjusted odds ratio [aOR] 1.84; 95% CI 1.11, 3.05), female sex (aOR = 3.05; 1.67, 5.58) and BMI (aOR = 1.53 per 5 units BMI; 1.10, 2.11). The AUC of the fully adjusted MTF prevalence model was 0.689 (95% CI 0.625, 0.754). Significant cross-sectional predictors of prevalent LTF tenderness included only female sex (aOR = 2.18; 1.22, 3.90). The AUC of the fully adjusted LTF prevalence model was 0.637 (95% CI 0.563, 0.711). There were no significant cross-sectional predictors of prevalent PF grind in the fully adjusted model. However, MPO was predictive of PF grind in age-sex-BMI adjusted models, and it remained borderline significant in the fully adjusted model. The AUC of the fully adjusted PF prevalence model was 0.625 (95% CI 0.538, 0.712).

**Table 3:** Cross-sectional (prevalence) model odds ratios with 95% confidence intervals

Variable	Age-sex-BMI adjusted	Fully adjusted^a^
Medial tibiofemoral tenderness models		
Female	2.24 (1.35, 3.72)^b^	3.05 (1.67, 5.58)
Age (per 5 years)	1.04 (0.92, 1.17)^b^	0.98 (0.85, 1.12)
BMI (per 5 units)	1.56 (1.18, 2.08)^b^	1.53 (1.10, 2.11)
Medial femoral cartilage	1.41 (1.06, 1.88)	1.84 (1.11, 3.05)
Medial tibial cartilage	1.19 (0.90, 1.58)	0.80 (0.49, 1.30)
Medial femoral osteophytes	1.08 (0.83, 1.41)	0.92 (0.60, 1.41)
Medial tibial osteophytes	1.13 (0.84, 1.51)	0.89 (0.54, 1.44)
Medial anterior meniscus	0.69 (0.20, 2.39)	0.31 (0.07, 1.30)
Medial meniscal body	1.79 (0.92, 3.51)	1.66 (0.70, 3.95)
Medial posterior meniscus	1.69 (0.92, 3.13)	1.31 (0.61, 2.82)
Lateral tibiofemoral tenderness models		
Female	2.04 (1.16, 3.57)^b^	2.18 (1.22, 3.90)
Age (per 5 years)	1.03 (0.91, 1.17)^b^	1.02 (0.88, 1.18)
BMI (per 5 units)	1.14 (0.88, 1.49)^b^	1.06 (0.80, 1.40)
Lateral femoral cartilage	0.84 (0.63, 1.13)	0.73 (0.50, 1.07)
Lateral tibial cartilage	1.00 (0.73, 1.38)	0.90 (0.52, 1.55)
Lateral femoral osteophytes	1.13 (0.86, 1.49)	1.00 (0.66, 1.52)
Lateral tibial osteophytes	1.23 (0.91, 1.67)	1.31 (0.82, 2.10)
Lateral anterior meniscus	1.14 (0.44, 3.01)	0.60 (0.14, 2.65)
Lateral meniscal body	1.87 (0.72, 4.86)	3.31 (0.61, 17.83)
Lateral posterior meniscus	1.44 (0.47, 4.43)	1.12 (0.28, 4.45)
Patellofemoral grind models		
Female	0.97 (0.53, 1.77)^b^	1.14 (0.60, 2.16)
Age (per 5 years)	0.90 (0.78, 1.05)^b^	0.87 (0.73, 1.03)
BMI (per 5 units)	1.04 (0.77, 1.40)^b^	0.89 (0.64, 1.24)
Patellar cartilage	1.04 (0.75, 1.45)	0.82 (0.54, 1.25)
Trochlear groove cartilage	1.13 (0.80, 1.60)	1.01 (0.65, 1.57)
Medial patellar osteophytes	1.67 (1.11, 2.51)	1.61 (0.94, 2.75)
Lateral patellar osteophytes	1.43 (0.99, 2.08)	1.29 (0.78, 2.14)
Superior patellar osteophytes	1.22 (0.81, 1.83)	0.88 (0.50, 1.55)
Inferior patellar osteophytes	1.27 (0.88, 1.84)	1.04 (0.62, 1.75)

^a^Fully adjusted medial tibiofemoral tenderness models include all MRI predictors listed in that section in addition to age, sex and BMI; fully adjusted lateral tibiofemoral tenderness models include all MRI predictors listed in that section in addition to age, sex and BMI; fully adjusted patellofemoral grind models include all MRI predictors listed in that section in addition to age, sex and BMI

^b^Age, sex and BMI effects listed in the age-sex-BMI adjusted column are unadjusted

Table 4 lists the longitudinal (incidence) model odds ratios with 95% CIs, both age-sex-BMI adjusted as well as fully adjusted. There were no significant predictors of 3-year incident MTF tenderness. The AUC of the fully adjusted MTF incidence model was 0.731 (95% CI 0.613, 0.849). Significant predictors of 3-year incident LTF tenderness included only female sex (aOR = 3.83; 1.25, 11.77). The AUC of the fully adjusted LTF incidence model was 0.756 (95% CI 0.641, 0.871). Significant predictors of 3-year incident PF grind included only LPO in the fully adjusted model (aOR = 4.82; 1.69, 13.77). However, in the age-sex-BMI adjusted model, PC was also a predictor. The AUC of the fully adjusted PF incidence model was 0.815 (95% CI 0.715, 0.915).

**Table 4:** Longitudinal (incidence) model odds ratios with 95% confidence intervals

Variable	Age-sex-BMI and individual follow-up time adjusted	Fully adjusted^a^
Medial tibiofemoral tenderness models		
Female	2.06 (0.75, 5.61)^b^	2.39 (0.78, 7.37)
Age (per 5 years)	1.17 (0.94, 1.46)^b^	1.14 (0.82, 1.57)
BMI (per 5 units)	1.23 (0.64, 2.35)^b^	1.18 (0.56, 2.50)
Medial femoral cartilage	1.35 (0.78, 2.34)	1.21 (0.46, 3.21)
Medial tibial cartilage	1.37 (0.84, 2.24)	1.25 (0.45, 3.43)
Medial femoral osteophytes	1.24 (0.68, 2.27)	0.89 (0.40, 1.98)
Medial tibial osteophytes	1.38 (0.71, 2.65)	1.01 (0.35, 2.92)
Medial anterior meniscus	2.58 (0.61, 10.83)	2.83 (0.36, 22.11)
Medial meniscal body	1.12 (0.37, 3.41)	0.66 (0.10, 4.25)
Medial posterior meniscus	1.21 (0.43, 3.39)	0.74 (0.14, 4.00)
Lateral tibiofemoral tenderness models		
Female	3.06 (1.09, 8.53)^b^	3.83 (1.25, 11.77)
Age (per 5 years)	1.12 (0.88, 1.42)^b^	1.13 (0.78, 1.64)
BMI (per 5 units)	1.28 (0.61, 2.68)^b^	1.53 (0.76, 3.09)
Lateral femoral cartilage	0.61 (0.29, 1.27)	0.61 (0.24, 1.56)
Lateral tibial cartilage	0.62 (0.30, 1.29)	0.67 (0.29, 1.56)
Lateral femoral osteophytes	0.62 (0.26, 1.48)	0.79 (0.27, 2.33)
Lateral tibial osteophytes	0.70 (0.26, 1.86)	0.98 (0.32, 3.06)
Lateral anterior meniscus	0.77 (0.11, 5.40)	0.79 (0.08, 8.21)
Lateral meniscal body	0.96 (0.18, 5.02)	3.59 (0.30, 42.59)
Lateral posterior meniscus	0.72 (0.13, 4.05)	1.79 (0.23, 13.85)
Patellofemoral grind models		
Female	1.66 (0.58, 4.69)^b^	1.87 (0.62, 5.68)
Age (per 5 years)	0.89 (0.66, 1.21)^b^	0.69 (0.44, 1.06)
BMI (per 5 units)	1.04 (0.55, 1.94)^b^	1.12 (0.52, 2.42)
Patellar cartilage	1.72 (1.08, 2.75)	1.18 (0.58, 2.42)
Trochlear groove cartilage	1.56 (0.98, 2.51)	1.14 (0.53, 2.42)
Medial patellar osteophytes	1.70 (0.88, 3.30)	1.40 (0.55, 3.54)
Lateral patellar osteophytes	5.40 (2.13, 13.68)	4.82 (1.69, 13.77)
Superior patellar osteophytes	0.99 (0.50, 1.94)	n/a^c^
Inferior patellar osteophytes	0.93 (0.40, 2.21)	0.45 (0.16, 1.28)

^a^Fully adjusted medial tibiofemoral tenderness models include all MRI predictors listed in that section in addition to age, sex, BMI and individual follow-up time; fully adjusted lateral tibiofemoral tenderness models include all MRI predictors listed in that section in addition to age, sex, BMI and individual follow-up time; fully adjusted patellofemoral grind models include all MRI predictors listed in that section in addition to age, sex, BMI and individual follow-up time

^b^Age, sex and BMI effects listed in the age-sex-BMI adjusted column are unadjusted

^c^Superior patellar osteophytes was omitted from the fully adjusted 3-year incident patellofemoral grind model due to nonconvergence

Finally, in sensitivity analyses, we found a significant cross-sectional positive association between PF grind and LFO (aOR = 2.41; 1.42, 4.10), but this was offset by a negative association with LTO in the same model (aOR = 0.51; 0.29, 0.90), indicating potential problems with fit.

## Discussion

We have explored potential MRI predictors (cartilage, osteophytes and meniscus) of prevalent and 3-year incident medial and lateral tibiofemoral knee joint tenderness and patellofemoral grind. Significant predictors of prevalent MTF tenderness included medial femoral cartilage, female sex and BMI. Predictors of prevalent LTF tenderness included female sex. There were no predictors of prevalent PF grind in the fully adjusted model. However, medial patellar osteophytes was predictive in the age-sex-BMI adjusted model. There were no predictors of 3-year incident MTF tenderness. Predictors of 3-year incident LTF tenderness included female sex. Predictors of 3-year incident PF grind included lateral patellar osteophytes. In the age-sex-BMI adjusted model, patellar cartilage was also a predictor.

There have been relatively few studies investigating structural correlates of knee joint tenderness (on palpation) or PF grind, and the structural measures in these studies were generally aggregated. Parsons et al. for example published one such (cross-sectional) study, but considered only the aggregated structural measures “tibiofemoral OA” or “patellofemoral OA” evaluated on X-ray [7]. However, pertinent structural defects related to OA may occur in various specific structures (e.g., cartilage/osteophytes/meniscus) and within various specific subregions (medial/lateral, anterior/posterior, tibiofemoral/patellofemoral). The data utilized in our study include such specificity. Despite these differences, however, some comparisons may still be made to previous literature in this area. Parsons et al. investigated tibiofemoral tenderness and crepitus. They found a positive association between tibiofemoral OA and tenderness (OR = 7.8). While their reported effect is ostensibly higher than our estimated association between medial femoral cartilage grade and medial tibiofemoral tenderness (aOR = 1.84), our reported effect is *per grade* (on a 4-point scale). Appling our models to 3- and 4-point increases in medial femoral cartilage grade (obtained by exponentiation) result in similar estimates to that of Parsons et al. On the lateral side, however, we did not observe a significant adjusted association between MRI scores and lateral tibiofemoral tenderness. As Parsons et al. did not differentiate between medial and lateral symptoms or structural defects, it is unclear whether our lateral results contradict those data. Although Parsons et al. did not model patellofemoral grind per se, they did model crepitus. They found a significant association between crepitus and tibiofemoral OA (OR = 3.9), but no significant association with patellofemoral OA. Similarly, in our study, we did not find an association between PF grind and MRI measures of patellar or trochlear groove cartilage or osteophytes, and due to theoretical considerations (lack of a plausible biological pathway), we did not regress PF grind versus tibiofemoral MRI scores. However, in sensitivity analyses, we did find a significant cross-sectional association between PF grind and LFO (aOR = 2.41), albeit offset by a negative association with LTO in the same model (aOR = 0.51), indicating potential problems with fit. In another cross-sectional study of structural predictors of knee joint tenderness, Saitu et al. utilized ultrasound to assess structural defects, and fit logistic regression models to assess correlation with joint line tenderness (JLT) [19]. They found significant positive associations between JLT and female sex (OR = 11.87) as well as cartilage thickness (OR = 0.12). Female sex was also significantly predictive in both our cross-sectional tenderness models. Inverting their OR for cartilage thickness produces an OR = 8.33, which ostensibly appears higher than our corresponding OR of 1.84 for MTF tenderness vs. MFC. However, Saitu et al. measured cartilage thickness in mm, with an interquartile range (IQR) of just 0.5 mm, and a between-group difference in medians of just 0.2 mm, yet they reported their OR in a per-mm scale. As such, a more comparable estimate to our per-grade OR could be obtained by taking the fourth root of their reported OR, which yields an OR = 1.70, very close to ours. As in our study, Saitu et al. did not find an association between JLT and osteophytes in their overall sample (though they did observe an association in a selected pre-radiographic subsample). In another study, Wang et al. considered the same two outcome variables as our study, namely joint line tenderness and patellofemoral grind, albeit without differentiating medial and lateral tenderness [9]. They found that PF grind (but not tenderness) was associated with cartilage volume loss (assessed on MRI) cross-sectionally, which is the opposite of our finding in which cartilage defects predict tenderness but not PF grind.

Longitudinal studies predicting new onset knee joint tenderness or PF grind per se are fewer still in the existing literature, in fact we have not identified any such studies per se. In the closest such study (also cited above), Wang et al. studied “fluctuating” (present at only one of two time points) and “persistent” (present at each of two time points) patterns of knee joint tenderness and PF grind, versus *changes* in structural defects [9]. It is worth noting that the structural defects considered in that study were aggregated: “cartilage volume loss” (on MRI) and “worsening of radiographic osteoarthritis” (which they used to describe worsening osteophytes on X-ray). Wang et al. found no association between pattern of knee joint tenderness and change in structural defects (not exactly the same research question as ours in either the dependent or independent variables but nonetheless a similarly null finding). Wang et al. did find an association between pattern of PF grind and rate of cartilage loss over time, but again this is not identical to our research question.

The strengths and limitations of our study deserve comment. While population-based is a strength, the target population is not the general population, but those with baseline knee pain, aged 40–79 at baseline, who were followed up over an average of 7.5 years. However, considering our objective was to explore potential MRI predictors (cartilage, osteophytes and meniscus) of prevalent and 3-year incident medial and lateral tibiofemoral knee joint tenderness and patellofemoral grind, symptoms of an inherently painful disease (OA), this restriction should not be too impactful. Furthermore, our inclusion of mild but persistent knee pain without diagnosed OA may present opportunities to develop preemptive strategies aimed at reducing tenderness and grind both in the present and future, in those at risk of such symptoms (e.g., those with pre-radiographic OA). Another limitation of the models explored herein is that their application would require an MRI, which can be expensive. However, an associated strength of this study is precisely the fact that it is based on MRI data, and as such covers a wide range of specific structures (e.g., cartilage/osteophytes/meniscus), various specific compartments (medial/lateral, anterior/posterior, tibiofemoral/patellofemoral/trochlear groove), and up to four scoring levels per compartment. Another important limitation is the relatively small sample size utilized in this study: the baseline sample (*N* = 255) for cross-sectional modelling, and the intersection of the first and second follow-up cycles (*N* = 108 × 2 = 216) for longitudinal modelling. Future validation studies with larger data sets will be needed to confirm our findings.

We have explored potential MRI predictors (cartilage, osteophytes and meniscus) of prevalent and 3-year incident medial and lateral tibiofemoral knee joint tenderness and patellofemoral grind. Cross-sectionally, predictors of MTF tenderness included MFC, female sex and BMI, but nothing predicted 3-year incidence. Female sex alone predicted cross-sectional and longitudinal LTF tenderness. While nothing predicted PF grind in fully adjusted cross-sectional models, LPO was a predictor of 3-year incidence. These findings could potentially help to guide preemptive strategies aimed at reducing tenderness and grind both in the present and future (3-year incidence), two common symptoms of osteoarthritis.

## Data Availability

The datasets used and analyzed during the current study are available from the corresponding author on reasonable request.
